# The Influence of Transgenic Maize on the Endophytic Microorganisms of *Eisenia fetida*

**DOI:** 10.3390/microorganisms14020302

**Published:** 2026-01-28

**Authors:** Xinyao Xia, Shuke Yang, Xue Song, Chaofeng Hao, Hongwei Sun, Xiaohui Xu, Xingbo Lu, Fan Li

**Affiliations:** 1 Shandong Key Laboratory for Green Prevention and Control of Agricultural Pests, Institute of Plant Protection, Shandong Academy of Agricultural Sciences, Jinan 250100, China; 2 Key Laboratory for Safety Assessment (Environment) of Agricultural Genetically Modified Organisms, Ministry of Agriculture and Rural Affairs, Jinan 250100, China; 3 The Engineering Research Institute of Agriculture and Forestry, College of Horticulture, Ludong University, 186 Hongqizhong Road, Yantai 264025, China

**Keywords:** *Eisenia fetida*, transgenic maize, microbiota

## Abstract

To evaluate the comprehensive ecological risks associated with transgenic plant residues, this study examined their impact on *Eisenia fetida* and their endogenous microorganisms. The results indicated that transgenic plant residues did not influence the survival or weight of *E. fetida*, but they significantly altered the microbial community structure at specific time points. Specifically, the diversity and structure of the fungal community exhibited significant changes on the 14th and 28th days after treatment. In contrast, the bacterial response was delayed, with 22 biomarkers, including *Caproiciproducens*, *Lachnoclostridium*, and *Enterococcus*, being specifically enriched on the 21st day. This study confirmed that transgenic plant residues can temporally reshape the microecology within *E. fetida*. The practical significance of this research lies in highlighting the importance of incorporating the microbiome into safety assessment frameworks, thereby providing a scientific foundation for developing more forward-looking ecological risk assessment standards.

## 1. Introduction

With the global commercialization of genetically modified (GM) crops, concerns regarding their ecological safety have garnered significant international attention. One critical aspect of assessing the ecological safety of GM crops is their impact on non-target organisms [1]. During cultivation, genetically modified insect-resistant crops can introduce materials into the soil ecosystem through mechanisms such as pollen drift, residue deposition, root exudation, and the incorporation of crop residues [2]. Consequently, these crops may exert potential effects on non-target soil organisms. Earthworms, which constitute 60% to 80% of the total soil fauna, are integral to soil ecosystems due to their roles in organic matter decomposition, nutrient cycling, and soil structure enhancement [3]. Among earthworms, *E. fetida* is particularly sensitive to toxins and is frequently employed as an indicator species in soil ecological safety assessments [4]. *E. fetida* is recognized by the Organisation for Economic Co-operation and Development (OECD) as a standard model organism for soil ecotoxicological research, with established protocols for acute and subacute toxicity testing.

Traditional assessments of the effects of genetically modified insect-resistant crops on *E. fetida* predominantly emphasize phenotypic indicators such as survival rate, weight change, growth and development, and reproductive capacity [5]. Nevertheless, these macroscopic parameters often lack the sensitivity required to detect sub-lethal effects under conditions of low dose and prolonged exposure. The microbial community within an organism is a crucial indicator of host health, with alterations potentially manifesting prior to observable macroscopic biological changes, thereby serving as early warning indicators [5]. Due to its high sensitivity, functional correlation, and ecological indicator value, the microbial diversity within the intestinal and body cavity of earthworms has increasingly been recognized as an innovative biomarker for assessing the impact of genetically modified crops on these organisms [6]. The microbial community residing within the intestinal and body cavity of earthworms establishes a symbiotic relationship with the host, contributing to essential physiological processes such as food digestion (e.g., cellulose degradation), immune regulation (e.g., pathogen inhibition), and environmental toxin metabolism (e.g., heavy metal detoxification) [7]. This community’s structure is highly sensitive to alterations in both the internal and external environments of the host.

In this study, genetically modified insect-resistant corn plant tissues were incorporated into artificial soil under controlled indoor conditions to simulate the natural exposure and survival environment of *E. fetida*. By examining the variations in microbial diversity and community structure within *E. fetida* over different cultivation periods, this study assessed the ecological safety of genetically modified insect-resistant corn on non-target organisms, specifically *E. fetida.*

## 2. Materials and Methods

### 2.1. Genetically Modified Insect-Resistant and Herbicide-Tolerant Corn

The transgenic corn strain LD05, which harbors the bar and m2cryAb-vip3A genes, was selected as the experimental treatment material, while the non-genetically modified Zheng58 corn served as the control. Upon reaching the jointing stage, 150 g of leaves was harvested from each corn type, with this procedure being conducted on four separate occasions. The harvested leaves were immediately ground in the field and subsequently frozen using liquid nitrogen for preservation. Upon transport to the laboratory, the samples underwent freeze-drying for a duration of 24 h and were then stored at −80 °C for future analyses. Employing freeze-dried leaf material in the experiment is advantageous for maximizing the preservation of protein activity.

### 2.2. The Influence of Transgenic Plant Residues on Microbial Diversity of E. fetida

The artificial soil technique was employed to replicate natural exposure conditions. Freeze-dried leaf powder was incorporated into the sterile substrate (70% quartz sand, 20% kaolin clay, 10% sphagnum peat, adjusted to pH 6.0 ± 0.5) and thoroughly homogenized. The sterile substrate was obtained by high temperature sterilization at 121 °C for 30 min using a laboratory autoclave (Tomy autoclave SX-500, Tomy Kogyo Co., Ltd., Tokyo, Japan). Distilled water was subsequently added to adjust the soil moisture content to a range of 30% to 35%. A total of 500 g of the prepared soil, ensuring a soil layer thickness of no less than 8 cm in a 1000 mL beaker, was placed into the beaker, followed by the introduction of 10 *E. fetida*. The opening of the beaker was securely covered with gauze. The beaker was then positioned in an artificial climate chamber maintained at a temperature of (20 ± 2) °C, with relative humidity ranging from 80% to 85%, and exposed to continuous illumination with a light intensity of 400 lx to 800 lx.

Prior to the commencement of the experiment, the *E. fetida* specimens underwent a fasting period of 24 h, during which they were placed on moist filter paper. This procedure was implemented to evacuate their intestinal contents and minimize interference from environmental microorganisms. The body cavity fluid or intestinal contents were then extracted via dissection; it is noteworthy that some studies opted to homogenize the entire *E. fetida*, necessitating caution regarding potential soil residue contamination. At intervals of 7, 14, 21, and 28 days post cultivation, a single *E. fetida* was randomly selected, rapidly frozen in liquid nitrogen, and subsequently stored at −80 °C.

During the jointing stage, 150 g of corn leaves was collected from each plot. The leaves were frozen in liquid nitrogen in the field, then brought back to the laboratory and freeze-dried for 24 h. They were then stored at −80 °C for future use. The freeze-dried leaf powder was added to the artificial soil, and 10 *E. fetida* were placed in each treatment. The experiment was conducted in an artificial climate chamber. On the 7th and 14th days, the soil in the beakers was poured out, and the survival rate and weight of the *E. fetida* were observed and recorded.

### 2.3. DNA Extraction and Amplicon Sequencing

Extract the DNA from the *E. fetida* using the QIAGEN DNeasy Blood & Tissue Kit ((Qiagen, Hilden, Germany). Perform DNA quality testing using Qubit^®^ dsDNA HS Assay (Thermo Fisher Scientific, Waltham, MA, USA) and NanoDrop 2000c (Thermo Fisher Scientific, USA). Amplify the bacterial 16S rRNA gene using primers 341F (5′-CCTAYGGGRBGCASCAG-3′)/805R (5′-GACTACHVGGGTATCTAATCC-3′). Expand the fungal ITS region using primers ITS1F (5′-CTTGGTCATTTAGAGGAAGTAA-3′)/ITS2R (5′-GCTGCGTTCTTCATCGATGC-3′). The amplification reaction contains 15 μL KAPA HiFi HotStart ReadyMix (Roche, Basel, Switzerland), 2 μM primers, and 10 ng template DNA. Thermal cycling consisted of initial denaturation at 95 °C for 3 min, followed by 95 °C for 30 s, annealing at 55 °C for 30 s (for bacteria) or 50 °C for 30 s (for fungi), extension at 72 °C for 30 s (30 cycles), and final extension at 72 °C for 5 min. Construct the library using the Nextera XT Index Kit (Catalog no. FC-131-1096, Illumina, San Diego, CA,, USA) and quantify the library concentration using Qubit^®^. Ensure balanced sample concentrations. Confirm library fragment sizes using a Agilent 2100 Bioanalyzer (Santa Clara, CA, USA). After library construction, perform amplicon sequencing using the Illumina MiSeq (2 × 250/300 bp, Wekemo Tech Group Co., Ltd., Shenzhen, China) platform.

### 2.4. Bioinformatic Analysis of Amplicon Sequencing Data

The analysis of bacterial 16S rRNA genes and fungal ITS sequences was conducted using QIIME version 1.9 [8] and USEARCH version 10 [9]. Initially, FastQC version 0.11.5 [10] was employed to assess the quality of the reads. Subsequently, Trimmomatic version 0.39 [11] was utilized to trim paired reads with quality scores below Q30. The processed sequences were then clustered, with those exhibiting a similarity greater than 97% being classified as belonging to the same operational taxonomic unit (OTU). Taxonomic classification of the sequences was performed using the SILVA version 138.1 [12] and UNITE version 8.2 [13] databases to distinguish between bacterial and fungal sequences. For α diversity estimation, the OTU tables for bacteria and fungi were standardized using the non-GMOize_table.py script in QIIME, following the cumulative sum scaling (CSS) method. β diversity analysis was conducted using the beta_diversity.py script, also employing the CSS non-GMOization approach. Operational taxonomic units present in all samples were identified as core taxonomic units. Principal coordinates analysis (PCoA) was performed using the R software version 4.1.1 [14] environment, version 4.1.0, employing the Vegan package version 2.6.4 and the Tidyverse package version 2.0.0. PICRUSt2 [15] version 2.6.2 was used to perform functional prediction of 16S rRNA gene sequences in the Kyoto Encyclopedia of Genes and Genomes (KEGG) functional database. For the functional abundances among different samples, the G-TEST (for large samples: the number of annotated functional genes is greater than 20) and Fisher (for small samples: the number of annotated functional genes is less than 20) test methods in STAMP version 2.1.3 [16] were used to conduct pairwise significance difference tests between samples. A pairwise T-test was performed for different groups, with the *p*-value threshold set at 0.05 (values < 0.05 indicate significance). FAPROTAX version 1.2.3 [17] was used to perform the ecological function prediction.

## 3. Results

### 3.1. The Influence of Transgenic Plant Residues on the Body Weight and Survival Rate of E. fetida

Initially, we utilized corn leaves at the jointing stage, which was recognized as the tissue and organ exhibiting the highest level of exogenous protein expression throughout the growth period, as the experimental material. The genetically modified organism (GMO) was freeze-dried and subsequently added to the substrate soil for survival of *E. fetida*. This approach aimed to assess the impact of residual components from transgenic corn on the growth of *E. fetida.* The findings from a 14-day continuous experimental period indicated that, in comparison to the control group, transgenic plant residues did not exert a significant effect on the survival rate or weight change rate of *E. fetida* (Table 1).

### 3.2. The Basic Information of Amplicon Sequencing Data

**Figure 1:**
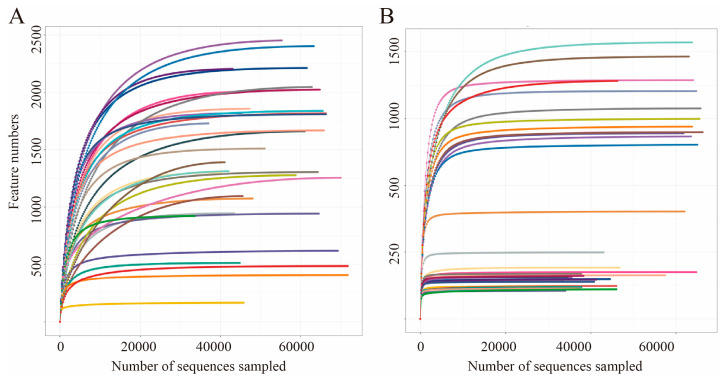
Rarefaction curves of 16S (**A**) and ITS (**B**) samples. Different colored curves correspond to different samples.

Transgenic plant residues were incorporated into artificial soil to replicate the natural exposure and survival conditions of *E. fetida*. On the 7th, 14th, 21st, and 28th days of cultivation, *E. fetida* specimens from both the treatment and control groups were randomly selected for further analysis. We characterized the bacterial and fungal community compositions by sequencing the small ribosomal subunit (16S rRNA) gene fragments and internally transcribed spacer (ITS) sequences across *Eisenia fetida*, followed by clustering into operational taxonomic units (OTUs) at 97% identity. From 32 samples, we obtained 1,764,191 high-quality rRNA reads and 1,612,640 ITS reads, with individual sample reads ranging from 33,638 to 71,951 and an average of 52,763 reads per sample, representing 28,460 bacterial and 10,457 fungal OTUs. The rarefaction curve, constructed based on the number of sequences and species, demonstrated a tendency to plateau, indicating that species richness in this environment does not significantly increase with additional sequencing, thereby satisfying the requirements for subsequent analysis (Figure 1).

### 3.3. The Influence of Transgenic Plant Residues on Microbial Alpha Diversity of E. fetida

**Figure 2:**
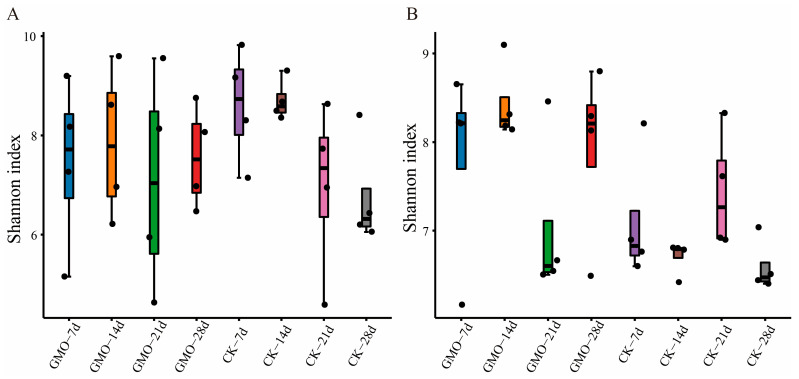
Shannon diversity index (SDI) of bacterial (**A**) and fungal communities (**B**).

Comparative analyses of Shannon diversity index (SDI) were conducted between GMO and non-GMO groups for both bacterial and fungal communities. The SDI for bacterial communities did not exhibit statistically significant differences between GMO and non-GMO groups across the four evaluated time points, as determined by the Wilcoxon rank-sum test (*p* > 0.05; Figure 2A, Table S1). In contrast, the SDI for fungal communities was observed to be lower on Days 14 and 28 in the non-GMO groups compared to the GMO group (*p* < 0.05; Figure 2B, Table S1). This finding suggests that the incorporation of transgenic plant residues may influence the diversity of fungal communities.

### 3.4. The Influence of Transgenic Plant Residues on Microbial Beta Diversity of E. fetida

**Figure 3:**
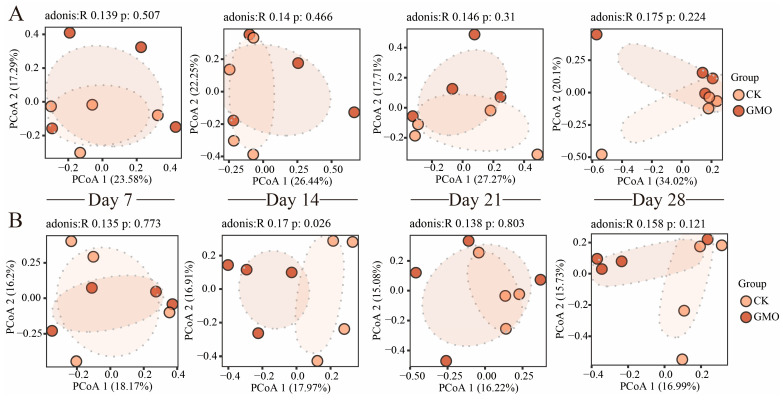
Principal coordinates analysis (PCoA) of bacterial (**A**) and fungal (**B**) communities based on Bray–Curtis distances among the four time points. Significant differences among the groups were determined using PERMANOVA (*n* = 32).

**Figure A1:**
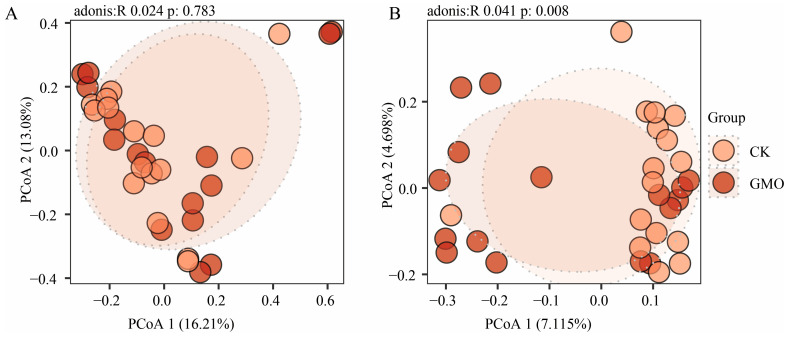
Principal coordinates analysis (PCoA) of bacterial (**A**) and fungal (**B**) communities based on Bray–Curtis distances between GMO and non-GMO groups. Significant differences among the groups were determined using PERMANOVA (*n* = 32).

Principal coordinates analysis (PCoA) utilizing Bray–Curtis dissimilarity effectively distinguished between the GMO and non-GMO groups in fungal communities (Figure A1B, Adonis R^2^ = 0.041, *p* = 0.008), as opposed to bacterial communities (Figure A1A, Adonis R^2^ = 0.024, *p* = 0.783), along the first principal coordinate. Concurrently, significant compositional differences in fungal communities were observed between the GMO and non-GMO groups at the Day 14 time point along the first principal component (Figure 3). Apart from this specific time point, no significant differences were detected between the GMO and non-GMO groups concerning bacterial and fungal communities. These PCoA findings suggest that genetically modified corn leaves exert a subtle influence on the composition of microbiome communities.

### 3.5. The Influence of Transgenic Plant Residues Corn Leaves on Microbial Community Structure of E. fetida

**Figure 4:**
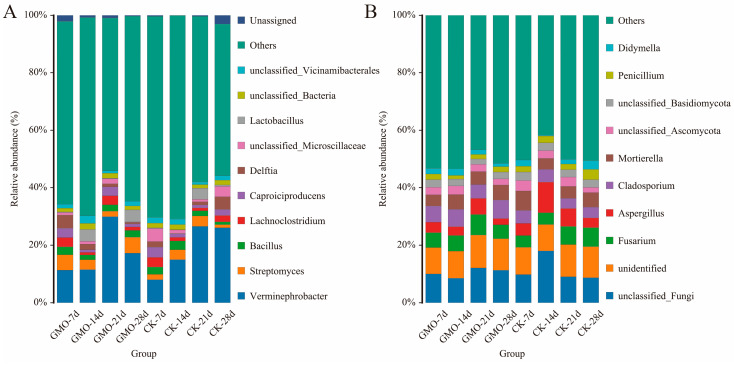
Relative abundance of the dominant bacterial (**A**) and fungal (**B**) genus between GMO and non-GMO groups among the 4 time points.

**Figure A2:**
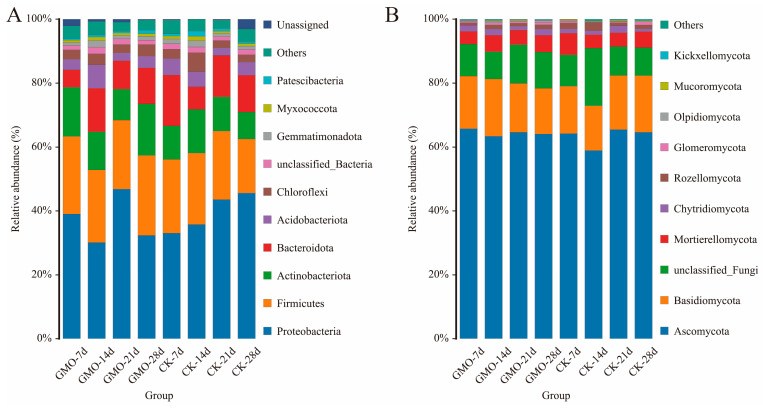
Relative abundance of the dominant bacterial (**A**) and fungal (**B**) phyla between GMO and non-GMO groups among the 4 time points.

To characterize the core microbiota associated with the GMO group, we conducted a comprehensive analysis of the taxonomic compositions and relative abundances of their bacterial and fungal communities. Analysis of microbial communities at the phylum level revealed similar compositions between the GMO and non-GMO groups (Figure A2A). The predominant bacterial phyla identified were *Proteobacteria* (38.27%), *Firmicutes* (22.20%), *Actinobacteriota* (12.06%), and *Bacteroidota* (10.85%). A parallel analysis of fungal communities indicated that *Ascomycota* (63.89%) and *Basidiomycota* (15.93%) were the dominant phyla (Figure A2B). At the genus level, the principal bacterial taxa included *Verminephrobacter* (19.20%), *Streptomyces* (3.27%), Bacillus (2.17%), and *Lachnoclostridium* (2.05%). The dominant fungal genera were *Fusarium* (5.46%), *Aspergillus* (4.88%), *Cladosporium* (4.87%), and *Mortierella* (4.83%) (Figure 4). According to the Wilcoxon rank-sum test, significant differences in relative abundance between the GMO and non-GMO groups were observed only for *Entotheonellaeota* and *Elusimicrobiota* at the 21-day time points (*p* < 0.05, Table S2). At the genus level, 12, 18, 15, and 19 taxa showed significant relative abundance differences between the GMO and non-GMO groups at the four time points, respectively (*p* < 0.05, Table S3). It is worth noting that no genus was continuously and significantly affected across the four time points.

### 3.6. The Influence of Transgenic Plant Residues on Microbial Biomarkers of E. fetida

**Figure 5:**
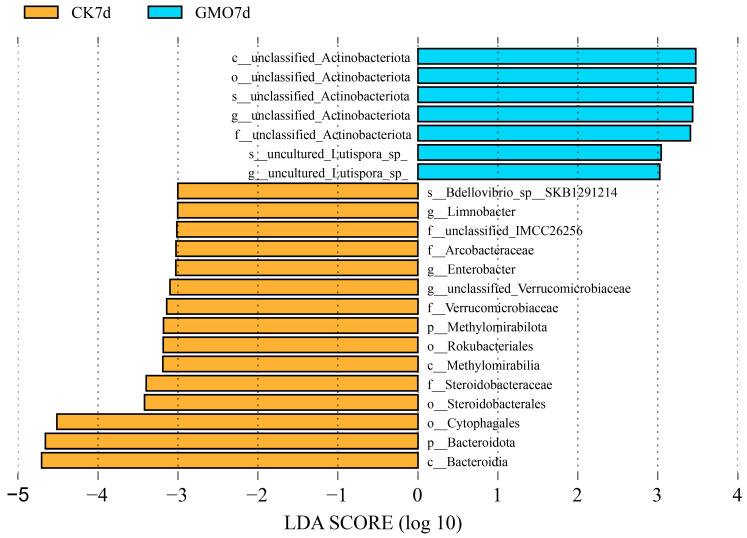
Biomarkers identified by LEfSe (LDA > 3) between GMO and the non-GMO maize groups at 7-day time point.

**Figure A3:**
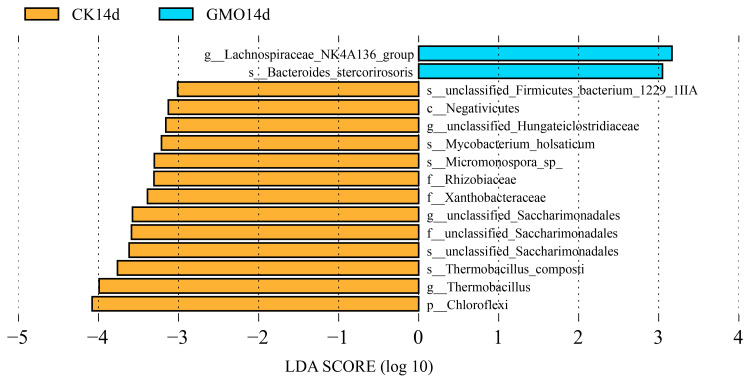
Biomarkers identified by LEfSe (LDA > 3) between GMO and the non-GMO maize groups at 14-day time point.

**Figure A4:**
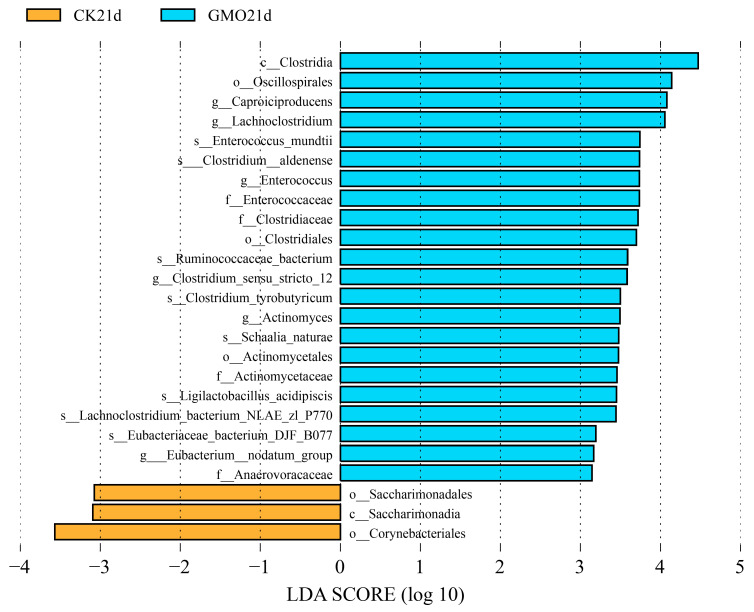
Biomarkers identified by LEfSe (LDA > 3) between GMO and non-GMO maize groups at 21-day time point.

**Figure A5:**
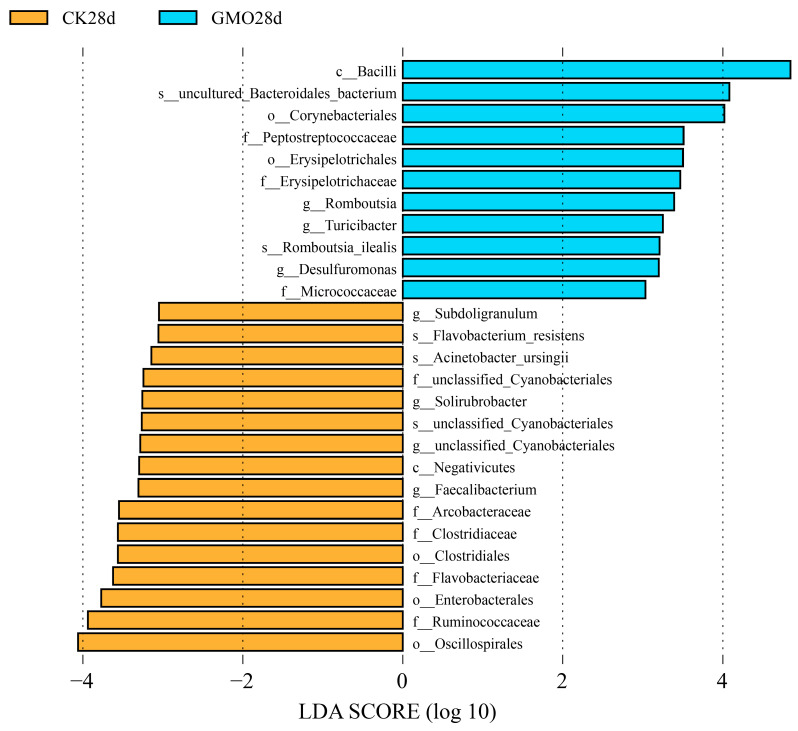
Biomarkers identified by LEfSe (LDA > 3) between GMO and non-GMO maize groups at 28-day time point.

Line Discriminant Analysis (LDA) Effect Size (LEfSe) is an analytical technique that integrates the non-parametric Kruskal–Wallis and Wilcoxon rank-sum tests with the effect size derived from LDA. This method is employed to identify biomarkers exhibiting statistically significant differences across various groups. As illustrated in Figure 5, at the 7-day time point, the GMO group exhibited 7 unclassified taxa as biological marker genera, whereas the non-GMO group presented 15 biological markers, including *Limnobacter* and *Enterobacter* (Figure 5). At the 14-day time point, only 2 biomarkers, *Lachnospiraceae_NK4A136_group* and *Bacteroides stercorirosoris*, were identified in the GMO group, while the non-GMO group displayed 13 biological markers (Figure A3). Notably, 22 biological markers, such as *Caproiciproducens*, *Lachnoclostridium*, and *Enterococcus*, were identified, with only 2 biological markers found in the non-GMO group (Figure A4). At the 28-day time point, 11 biological markers were identified in the GMO group, and 16 were identified in the non-GMO group (Figure A5). Noteworthy changes in the quantity of biological markers were observed on the 21st day following the introduction of transgenic leaves, indicating significant alterations in the microbial community within *E. fetida*.

### 3.7. The Influence of Transgenic Plant Residues on Microbial Function of E. fetida

To explore the functional disparities among microbial groups, we employed PICRUSt2 to predict 16S rRNA gene sequences within the KEGG database. Subsequently, we conducted pairwise significance tests between samples using the G-TEST and Fisher test methods in STAMP. The results showed that 2 and 20 KEGG functional terms showed significant enrichment differences between the GMO and non-GMO groups at the 7-day and 28-day time points, respectively. After treatment with transgenic plant residues for 2 h, AMPK signaling pathway and cyanoamino acid metabolism were enriched in the GMO group (Table S4). Regarding the 28-day time points, inositol phosphate metabolism, the RIG-I-like receptor signaling pathway, methane metabolism, steroid biosynthesis and degradation, retinol metabolism, and the HIF-1 signaling pathway and proteasome pathways were enriched in the GMO group (Table S7). There was no KEGG functional term for which enrichment difference was found at the 14-day and 21-day time points (Tables S5 and S6).

**Figure 6:**
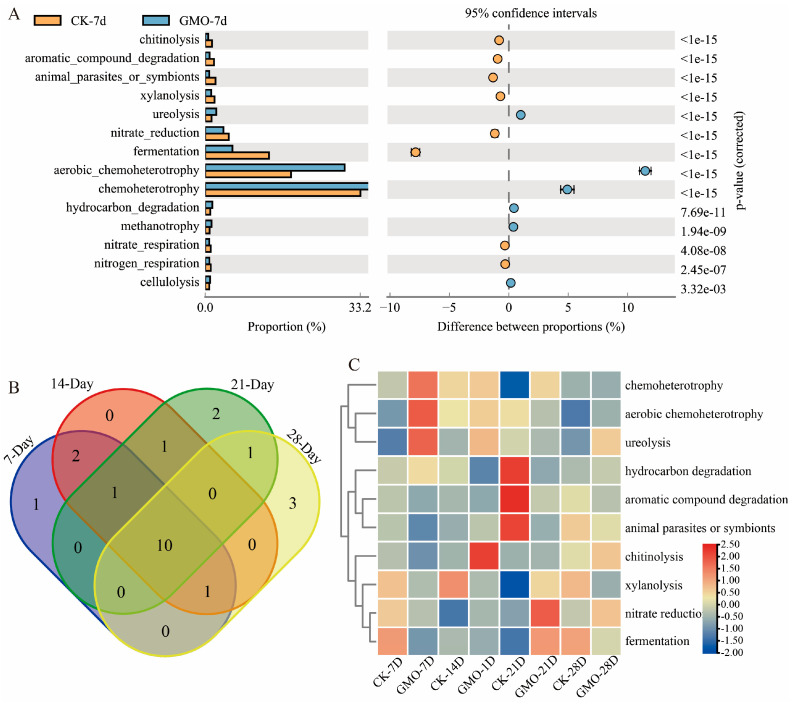
Inter-group ecological functional differences analysis. (**A**) Ecological function difference histogram between GMO and non-GMO groups at 7-day time point. Different colors represent different groups. The left image shows the abundance ratios of different functions in the two groups. The middle image represents the difference ratios of function abundances within the 95% confidence interval. The blue circles indicate that the GMO group is enriched in this ecological functional term, and the yellow circles indicate that the non-GMO group is enriched in this ecological functional term. The value on the rightmost side is *p*-values obtained from the pairwise T-test comparisons between different groups. (**B**) The shared ecological functional terms across all the four time points. (**C**) Heatmap of the abundance of the 10 shared ecological functional terms across the four time points.

**Figure A6:**
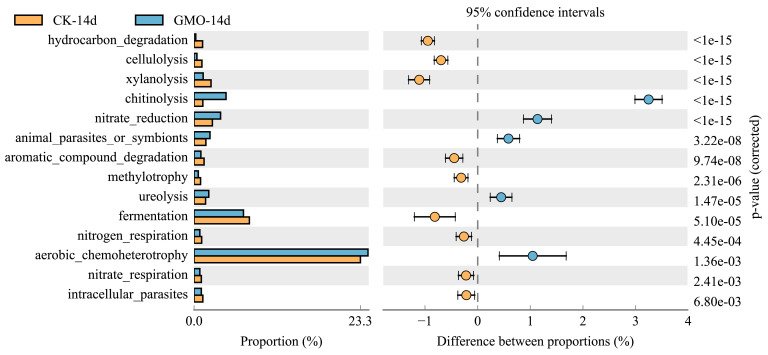
Ecological function difference histogram between GMO and non-GMO groups at 14-day time point. Different colors represent different groups. The left image shows the abundance ratios of different functions in two groups. The middle image represents the difference ratios of function abundances within the 95% confidence interval. The blue circles indicate that the GMO group is enriched in this ecological functional term, and the yellow circles indicate that the non-GMO group is enriched in this ecological functional term. The values on the rightmost side are *p*-values obtained from the pairwise T-test comparisons between different groups.

**Figure A7:**
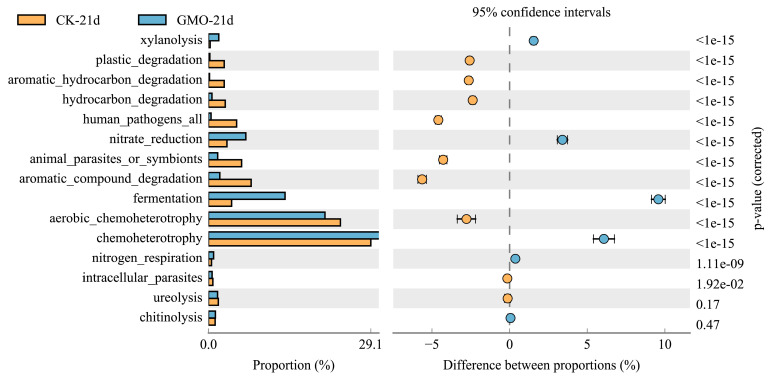
Ecological function difference histogram between GMO and non-GMO groups at 21-day time point. Different colors represent different groups. The left image shows the abundance ratios of different functions in the two groups. The middle image represents the difference ratios of function abundances within the 95% confidence interval. The blue circles indicate that the GMO group is enriched in this ecological functional term, and the yellow circles indicate that the non-GMO group is enriched in this ecological functional term. The values on the rightmost side are *p*-values obtained from the pairwise T-test comparisons between different groups.

**Figure A8:**
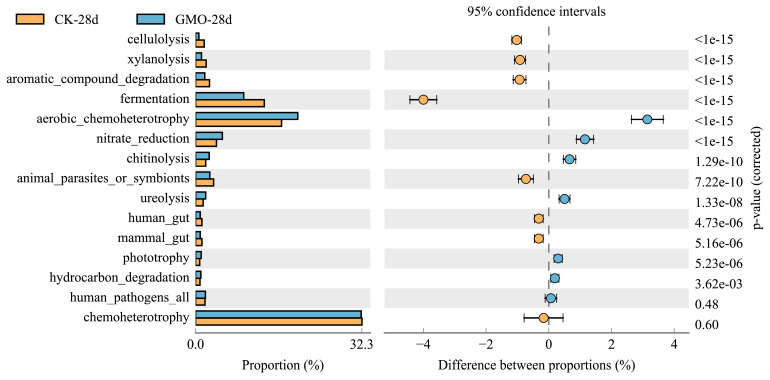
Ecological function difference histogram between GMO and non-GMO groups at 28-day time point. Different colors represent different groups. The left image shows the abundance ratios of different functions in two groups. The middle image represents the difference ratios of function abundances within the 95% confidence interval. The blue circles indicate that the GMO group is enriched in this ecological functional term, and the yellow circles indicate that the non-GMO group is enriched in this ecological functional term. The values on the rightmost side are *p*-values obtained from the pairwise T-test comparisons between different groups.

In addition, the ecological function (in particular the cycles of elements such as carbon, hydrogen, nitrogen, phosphorus, and sulfur) were predicted based on the published verified literature of culturable bacteria (Figure 6A, Figure A6, Figure A7 and Figure A8). There were 10 ecological functional terms that showed enrichment differences among all the four time points, including nitrate reduction, aromatic compound degradation, aerobic chemoheterotrophy, chitinolysis, animal parasites or symbionts, chemoheterotrophy, ureolysis, hydrocarbon_degradation, xylanolysis, and fermentation (Figure 6B). Among them, the aromatic compound degradation term was suppressed in the GMO group across the four time points. In contrast, the nitrate reduction, aerobic chemoheterotrophy, chitinolysis, chemoheterotrophy, and ureolysis terms were enriched in the GMO group at three out of the four time points (Figure 6C). Overall, transgenic plant residues cause greater ecological functional disturbances than KEGG terms in the bacterial community of *E. fetida*.

## 4. Discussion

The findings of this study suggest that, while the leaves of genetically modified corn did not significantly impact the survival rate, body weight, or other macroscopic physiological parameters of *E. fetida*, they did exert a notable and time-dependent regulatory influence on the endogenous microbial community within *E. fetida*, particularly affecting the fungal community. The Shannon diversity index demonstrated a significant increase in fungal diversity at both 14 and 28 days, and Principal Coordinates Analysis (PCOA) indicated a significant alteration in the fungal community structure at 14 days. These results collectively underscore a central conclusion: the consumption of genetically modified corn constitutes a critical environmental stressor that drives the succession of fungal communities within *E. fetida*’s gut. From an ecological standpoint, increased diversity is typically viewed as indicative of enhanced ecosystem stability and functional redundancy [18].

In this context, genetically modified corn may serve as a disruptive element that alters the original microbial equilibrium within the earthworm’s body, thereby creating ecological niches for certain fungal groups that were previously less abundant or possessed specific degradation functions. This disruption facilitates the reassembly and increased diversity of the fungal community. Such interference may arise from the direct influence of exogenous gene expression products, such as Bt protein in genetically modified crops, or from subtle alterations in the chemical composition of crop residues, including lignin and cellulose content [19]. These changes subsequently affect the composition of substrates available as carbon sources and energy for fungi. It is noteworthy that alterations in community structure were observed at 14 days, while increased diversity was evident at both 14 and 28 days. This suggests that the initial phase was characterized by intense community restructuring, eventually leading to a new, more diverse stable state. This finding is of significant importance, as it suggests that genetically modified crops, even in the absence of observable host toxicity effects, can potentially exert a substantial ecological impact on key soil decomposers, such as earthworms, by altering the composition of their symbiotic microorganisms [20]. Microbial communities, particularly fungal communities, are essential for the decomposition of organic matter, nutrient acquisition, and immune regulation in earthworms [21]. Consequently, it is imperative to explore in depth whether this alteration in community structure might further influence the long-term health, reproductive capacity, or functional roles of earthworms within the geochemical cycle.

In contrast to the initial response observed within the fungal community, the LEfSe analysis of the bacterial community in this study identified a distinct response emerging at the 21-day mark. Notably, 22 significant bacterial biomarkers were detected in the transgenic corn treatment group, including genera such as *Caproiciproducens*, *Lachnoclostridium*, and *Enterococcus*. This specificity at the 21-day interval suggests that the endosymbiotic bacterial community in *E. fetida* exhibits a “lag effect” in response to transgenic corn, indicating a dynamic ecological succession that diverges from that of the fungal community. Functional analysis of these biomarkers may offer mechanistic insights into the potential impacts of transgenic crops. For instance, *Caproiciproducens* is recognized for its ability to produce short-chain fatty acids, such as caproic acid, via the butyrate fermentation pathway, which are crucial regulators of host intestinal health [22]. Additionally, many members of the *Lachnoclostridium* genus participate in the degradation of cellulose and the fermentation of plant polysaccharides [23]. Enterococcus, commonly known as intestinal cocci, is typically regarded as an opportunistic pathogen; however, certain strains also exhibit probiotic properties [24]. The concurrent presence of these biomarkers strongly indicates that the consumption of transgenic corn may have altered the metabolic environment within the earthworm’s intestine. This alteration likely selectively enriches bacterial groups capable of more effectively utilizing transgenic corn residues as substrates or adapting to the novel chemical microenvironment within the intestinal tract. Specifically, transgenic corn may introduce unique carbohydrate structures that promote the proliferation of degradation bacteria such as *Lachnoclostridium*, whose metabolites subsequently create favorable conditions for *Caproiciproducens* and other acid-producing bacteria. This “relay-like” microbial cooperation may ultimately alter the spectrum of metabolic products within *E. fetida*’s intestine. Nonetheless, the potential benefits and drawbacks of this transformation warrant careful evaluation. On one hand, it may enhance *E. fetida*’s efficiency in decomposing specific plant materials; on the other hand, the enrichment of opportunistic pathogens like Enterococcus could pose health risks to *E. fetida* under certain environmental conditions. Therefore, the 22 biomarkers discovered in this study are not only indicators of the response but also a valuable starting point for subsequent research on their specific functions, interspecies interactions, and effects on the host physiology.

Based on the findings of this study, we can formulate a preliminary comprehensive assessment: The genetically modified corn examined herein functions as a “sub-lethal effect” stressor for *E. fetida*. While it does not induce direct toxicity at the individual level, as evidenced by survival and weight metrics, it prompts significant and temporally distinct alterations in the microbial community. Specifically, the fungal community exhibits initial changes in structure and diversity, whereas the bacterial community demonstrates specific group enrichment at a subsequent stage. This observed “microbial community priority” response pattern underscores the importance of integrating microbial ecological indicators into standard detection protocols when evaluating the environmental safety of genetically modified crops. Traditional acute toxicity assessments may fail to capture such profound and potentially enduring ecological impacts. Nonetheless, caution is warranted when interpreting these findings in the context of ecological risks. The observed alterations in microbial communities are neutral in themselves, and their ecological implications hinge on whether these changes adversely affect the host’s health or disrupt key ecological functions [25,26].

This study reveals a significant impact of transgenic plant residues on the ecological functions of the microbial communities associated with *E. fetida*. The most notable finding is that the transgenic treatment continuously inhibited the microbial degradation function of aromatic compounds, while promoting nitrate reduction, autotrophic oxidation energy metabolism, chitin decomposition, chemosynthetic metabolism, and urea decomposition at most time points. These results indicate that the addition of transgenic plant residues does not merely cause general disturbances to the microbial community structure but specifically alters the key functional patterns closely related to soil element cycling and organic matter transformation. From an ecological perspective, the continuous inhibition of the degradation pathways of aromatic compounds may imply that certain compounds (such as expressed proteins or their metabolites) in the transgenic residues interfere with the decomposition process of complex aromatic substances by microorganisms [27]. This may affect the natural degradation efficiency of organic pollutants and the humification process in the soil. Conversely, the enhancement of processes such as nitrate reduction and urea decomposition suggest that the transgenic residues may have promoted the microbial-driven nitrogen transformation function, possibly through providing differentiated carbon/nitrogen substrates or altering the microenvironmental conditions [28]. Such changes in nitrogen metabolic activity, if sustained over time, may have potential impacts on soil nitrogen availability and nitrogen gas emissions. Additionally, the enhancement of chitin decomposition ability may reflect a change in the microbial utilization strategy for chitin-like substances in the soil or in the intestinal tract of *E. fetida*. In conclusion, this study demonstrates that the residues of genetically modified corn have exerted non-neutral and directionally specific disturbances on the ecological functions of the earthworm microbiome. These disturbances show a certain degree of persistence over time. This provides a new functional perspective for understanding the potential impact of genetically modified crops on soil ecosystems.

## Figures and Tables

**Table 1:** The influence of transgenic plant residues on the body weight and survival rate of *E. fetida*.

Treatments		Survival Rate (%)	Body Weight Changes (%)
Day 7	GMO	92.50 ± 9.57 a	21.27 ± 1.09 a
	Non-GMO	97.50 ± 5.00 a	18.44 ± 10.48 a
Day 14	GMO	92.50 ± 9.57 a	28.49 ± 7.89 a
	Non-GMO	92.50 ± 9.57 a	30.10 ± 12.29 a

Wilcoxon rank-sum test was used to test for the significance of the difference between the two groups. The same letters indicate that there is no significant difference between the two groups (*p* > 0.05, *n* = 10).

## Data Availability

The original contributions presented in this study are included in the article and Supplementary Materials. Further inquiries can be directed to the corresponding author.

## Supplementary Materials

The following supporting information can be downloaded at: https://www.mdpi.com/article/10.3390/microorganisms14020302/s1, Table S1: Pairwise comparison of significant differences analysis between GMO and normal groups in both bacterial and fungal communities with Wilcoxon rank sum test; Table S2: *p* values of Wilcoxon rank sum test between the GMO and normal group at the phylum level at four time-points; Table S3: *p* values of Wilcoxon rank sum test between the GMO and normal group at the genus level at four time-points; Table S4: Bacterial function differences of *E. fetida* at 7-day time points; Table S5: Bacterial function differences of *E. fetida* at 14-day time points; Table S6: Bacterial function differences of *E. fetida* at 21-day time points; Table S7: Bacterial function differences of *E. fetida* at 28-day time points.

## References

[1] Carstens K., Anderson J., Bachman P., De Schrijver A., Dively G., Federici B., Hamer M., Gielkens M., Jensen P., Lamp W. (2012). Genetically modified crops and aquatic ecosystems: Considerations for environmental risk assessment and non-target organism testing. Transgenic Res..

[2] Lebedev V., Lebedeva T., Tikhonova E., Shestibratov K. (2022). Assessing impacts of transgenic plants on soil using functional indicators: Twenty years of research and perspectives. Plants.

[3] Akhila A., Entoori K. (2022). Role of earthworms in soil fertility and its impact on agriculture: A review. Int. J. Fauna Biol. Stud..

[4] Vannuccini M.L., Assenza R., Sturba L., Faleri C., Corsi I. (2025). Ecological risk assessment of sewage sludge as soil amendment: Lethal and sublethal effects in *Eisenia fetida*. Environ. Sci. Pollut. Res..

[5] Dwivedi S.A., Pandit T.R. (2024). Host Plant Resistance and Sustainable Management of Insect Pests. Antimicrobial Resistance in Agriculture and its Consequences.

[6] Solé M. (2020). Biomarkers in earthworms. Interaction and Fate of Pharmaceuticals in Soil-Crop Systems: The Impact of Reclaimed Wastewater.

[7] Medina-Sauza R.M., Álvarez-Jiménez M., Delhal A., Reverchon F., Blouin M., Guerrero-Analco J.A., Cerdán C.R., Guevara R., Villain L., Barois I. (2019). Earthworms building up soil microbiota, a review. Front. Environ. Sci..

[8] Caporaso J.G., Kuczynski J., Stombaugh J., Bittinger K., Bushman F.D., Costello E.K., Fierer N., Gonzalez Peña A., Goodrich J.K., Gordon J.I. (2010). QIIME allows analysis of high-throughput community sequencing data. Nat. Methods.

[9] Rognes T., Flouri T., Nichols B., Quince C., Mahé F. (2016). VSEARCH: A versatile open source tool for metagenomics. PeerJ.

[10] Brown J.W., Pirrung M., McCue L.A. (2017). FQC Dashboard: Integrates FastQC results into a web-based, interactive, and extensible FASTQ quality control tool. Bioinformatics.

[11] Bolger A.M., Lohse M., Usadel B. (2014). Trimmomatic: A flexible trimmer for Illumina sequence data. Bioinformatics.

[12] Quast C., Pruesse E., Yilmaz P., Gerken J., Schweer T., Yarza P., Peplies J., Glöckner F.O. (2012). The SILVA ribosomal RNA gene database project: Improved data processing and web-based tools. Nucleic Acids Res..

[13] Abarenkov K., Nilsson R.H., Larsson K.-H., Alexander I.J., Eberhardt U., Erland S., Høiland K., Kjøller R., Larsson E., Pennanen T. (2010). The UNITE database for molecular identification of fungi—Recent updates and future perspectives. New Phytol..

[14] Chambers J.M. (2008). Software for Data Analysis: Programming with R.

[15] Douglas G.M., Maffei V.J., Zaneveld J.R., Yurgel S.N., Brown J.R., Taylor C.M., Huttenhower C., Langille M.G.I. (2020). PICRUSt2 for prediction of metagenome functions. Nat. Biotechnol..

[16] Parks D.H., Tyson G.W., Hugenholtz P., Beiko R.G. (2014). STAMP: Statistical analysis of taxonomic and functional profiles. Bioinformatics.

[17] Sansupa C., Wahdan S.F.M., Hossen S., Disayathanoowat T., Wubet T., Purahong W. (2021). Can we use functional annotation of prokaryotic taxa (FAPROTAX) to assign the ecological functions of soil bacteria?. Appl. Sci..

[18] Biggs C.R., Yeager L.A., Bolser D.G., Bonsell C., Dichiera A.M., Hou Z., Keyser S.R., Khursigara A.J., Lu K., Muth A.F. (2020). Does functional redundancy affect ecological stability and resilience? A review and meta-analysis. Ecosphere.

[19] Abbas M.S.T. (2018). Genetically engineered (modified) crops (*Bacillus thuringiensis* crops) and the world controversy on their safety. Egypt. J. Biol. Pest Control.

[20] Powell J.R., Levy-Booth D.J., Gulden R.H., Asbil W.L., Campbell R.G., Dunfield K.E., Hamill A.S., Hart M.M., Lerat S., Nurse R.E. (2009). Effects of genetically modified, herbicide-tolerant crops and their management on soil food web properties and crop litter decomposition. J. Appl. Ecol..

[21] Doube B., Brown G. (2004). Functional interactions between earthworms, microorganisms, organic matter, and plants. Earthworm Ecology.

[22] Markowiak-Kopeć P., Śliżewska K. (2020). The Effect of probiotics on the production of short-chain fatty acids by human intestinal microbiome. Nutrients.

[23] Wu B., Ren T., Cao X., Wu T., Hu Z., Ai J., Zhang N., Zhang Y., Yu Z., Du L. (2025). Emerging and innovative utilisation of herbal medicine residues in anaerobic fermentation of corn straw: Cellulose degradation, fermentation characteristics, and microbial community structure and co-occurrence network. Ind. Crop. Prod..

[24] Krawczyk B., Wityk P., Gałęcka M., Michalik M. (2021). The Many Faces of *Enterococcus* spp.—Commensal, probiotic and opportunistic pathogen. Microorganisms.

[25] Girvan M.S., Campbell C.D., Killham K., Prosser J.I., Glover L.A. (2005). Bacterial diversity promotes community stability and functional resilience after perturbation. Environ. Microbiol..

[26] Martin L.B., Hopkins W.A., Mydlarz L.D., Rohr J.R. (2010). The effects of anthropogenic global changes on immune functions and disease resistance. Ann. N. Y. Acad. Sci..

[27] Fuchs G., Boll M., Heider J. (2011). Microbial degradation of aromatic compounds—From one strategy to four. Nat. Rev. Microbiol..

[28] Zhou S., Song Z., Li Z., Qiao R., Li M., Chen Y., Guo H. (2022). Mechanisms of nitrogen transformation driven by functional microbes during thermophilic fermentation in an ex situ fermentation system. Bioresour. Technol..

